# Time Series Data Fusion Based on Evidence Theory and OWA Operator

**DOI:** 10.3390/s19051171

**Published:** 2019-03-07

**Authors:** Gang Liu, Fuyuan Xiao

**Affiliations:** School of Computer and Information Science, Southwest University, Chongqing 400715, China; lg645187984@email.swu.edu.cn

**Keywords:** time series, data fusion, credibility decay model, OWA, target recognition

## Abstract

Time series data fusion is important in real applications such as target recognition based on sensors’ information. The existing credibility decay model (CDM) is not efficient in the situation when the time interval between data from sensors is too long. To address this issue, a new method based on the ordered weighted aggregation operator (OWA) is presented in this paper. With the improvement to use the Q function in the OWA, the effect of time interval on the final fusion result is decreased. The application in target recognition based on time series data fusion illustrates the efficiency of the new method. The proposed method has promising aspects in time series data fusion.

## 1. Introduction

Evidence theory, also called Dempster Shafer evidence theory [1,2], plays an important role in data fusion because of the efficiency to deal with uncertainty [3,4,5,6,7]. It is widely used in many applications, such as fault diagnoses [8,9,10,11], project assessment [12,13,14,15,16], risk management [17,18,19], target recognition [20,21,22,23,24,25], decision making [26,27,28,29] and so on [30,31,32,33,34].

Recently, besides uncertainty [35,36,37] and entropy [38,39,40,41,42] about evidence theory is well developed, how to take time series into consideration [43,44,45,46] and how to make a good data fusion [47,48,49] for different questions has received much attention. After many methods have been presented to deal with information statically, Smets [44] proposed a time decay model to combine information dynamically. Based on that model, Song et al. [43] reduced the effect of previous evidences through the discounting function in credibility decay model (CDM).

However, CDM has some shortcomings. For example, information collected from sensors may be unreasonably discounted in the CDM model when the time interval between two time nodes is relatively long. Simply dismissing the question may cause decisions to overly rely on the latest evidence without fully using existed collections of evidences and eventually lead to the wrong identification of the target. Additionally in CDM, there is also a lack of valid discussion on the relationship of time interval between old and new evidences and the credibility of old evidence.

To address this issue, a new method is proposed based on ordered weight aggregation (OWA) operator [50] to serve as a means redefining CDM. Past researches investigating OWA [51,52,53,54] showed the effectiveness of this operator in the information aggregation problem since OWA has the strength to easily adjust the degree of implicit “anding” and “oring” [50]. Based on time series, weights in OWA can be dynamically generated to effectively control the impact of old evidences on the final fusion result. The new decay model can identify the target using the information collected from sensors considering the role of old evidences in data fusion from the perspective of linguistic “anding” and “oring”, rather than time intervals, which is unreasonably used in original CDM.

The paper is arranged as follows. Section 2 introduces some preliminaries. Section 3 presents the new method to get discount for CDM based on OWA weights. Following that, two applications illustrate the performance of the proposed method in target recognition Section 4. Finally, a brief summary is given in Section 5.

## 2. Preliminaries

This section introduces some preliminary works including evidence theory, credibility decay model, and OWA.

### 2.1. Evidence Theory

It is inevitable to handle uncertainty in real applications [55,56,57], and transform complex situations into simple ones [58,59,60]. Evidence theory is effective in dealing with such a question [61,62,63,64,65] and other fields [66]. In the theory, a finite nonempty set with mutually exclusive and exhaustive attributes is called the frame of discernment, and denoted by Ω. The power set of Ω is $2^{\Omega }$, containing all subsets of Ω.

**Definition** **1.** *Let $\Omega =\{A_{1},A_{2},\cdots ,A_{n}\}$ be a frame of discernment. Assume A $\in 2^{\Omega }$. The basic probability distribution (BPA) of A or m(A) is a function defined as [1,2]:*

(1) m(A)→[0,1]

*and satisfies the following conditions:*

(2) $$m\left(\emptyset \right)=0\;\sum_{A\subseteq \Omega }m\left(A\right)=1$$

*If A=Ω and m(Ω)=1, we know nothing about the frame of discernment.*

One important advantage of evidence theory is that two BPAs can be combined together as follows.

**Definition** **2.** *Let $m_{1}$ and $m_{2}$ be two BPAs on *Ω*, and assume A,B,C are the subset of *Ω*. The Dempster combination rule, denoted by *⊕*, is defined as [1,2]:*

(3) $$\left(m_{1}⊕m_{2}\right)\left(A\right)=\left\{\begin{array}{cc} 0, & A=\emptyset \\ \frac{\sum_{B\cap C=A}m_{1}\left(B\right)m_{2}\left(C\right)}{1-\sum_{B\cap C=\emptyset }m_{1}\left(B\right)m_{2}\left(C\right)}, & A\neq \emptyset \end{array}\right.$$

Data fusion happens in constant combinations [67,68,69]. Conflict management [70,71] is an important part during combination [72,73,74]. Evidences from different sources are not totally reliable, credibility measures the reliability of evidence, which are defined as follows:

**Definition** **3.** *Let m be the BPA on the frame of discernment *Ω* with a credibility of α, then m could be discounted as [2]:*

(4) $$m^{\alpha }\left(A\right)=\left\{\begin{array}{cc} \alpha m(A), & A\neq \Omega \\ 1-\alpha +\alpha m(A), & A=\Omega \end{array}\right.$$

Although the unknownness of evidence increases by discounting the original BPA with credibility, the combination of evidence will be more effective due to the reduction of conflicts (Example 1).

**Example** **1.** *Assume a sensor S receives two evidence bodies $\left(E_{1},m_{1}\right)$, $\left(E_{2},m_{2}\right)$ on the discernment frame Ω={A,B,C}, which are shown as follows:*

$$\left(E_{1},m_{1}\right)=\left(\left[\left\{A\right\},0.99\right],\left[\left\{B\right\},0\right],\left[\left\{C\right\},0.01\right]\right)$$

$$\left(E_{2},m_{2}\right)=\left(\left[\left\{A\right\},0\right],\left[\left\{B\right\},0.99\right],\left[\left\{C\right\},0.01\right]\right)$$

*The combination results $m_{0}$ without credibility are as follows:*

$$m_{1}⊕m_{2}\left(A\right)=\frac{\sum_{E_{1}\cap E_{2}=A}m_{1}\left(E_{1}\right)m_{2}\left(E_{2}\right)}{1-\sum_{E_{1}\cap E_{2}=\emptyset }m_{1}\left(E_{1}\right)m_{2}\left(E_{2}\right)}=0$$

$$m_{1}⊕m_{2}\left(B\right)=\frac{\sum_{E_{1}\cap E_{2}=B}m_{1}\left(E_{1}\right)m_{2}\left(E_{2}\right)}{1-\sum_{E_{1}\cap E_{2}=\emptyset }m_{1}\left(E_{1}\right)m_{2}\left(E_{2}\right)}=0$$

$$m_{1}⊕m_{2}\left(C\right)=\frac{\sum_{E_{1}\cap E_{2}=A}m_{1}\left(E_{1}\right)m_{2}\left(E_{2}\right)}{1-\sum_{E_{1}\cap E_{2}=\emptyset }m_{1}\left(E_{1}\right)m_{2}\left(E_{2}\right)}=\frac{0.01\times 0.01}{1-(0.99\times 0.99+0.99\times 0.01+0.01\times 0.99)}=1$$

*Assume that the source of two evidence bodies $\left(E_{1},m_{1}\right)$ and $\left(E_{2},m_{2}\right)$, the sensor S, has only half the reliability, which means S is not fully trusted. So the discounted BPAs with half credibility (α=0.5) and combination results are shown as follows:*

$$\left(E_{1},m_{1}^{\alpha }\right)=\left(\left[\left\{A\right\},0.495\right],\left[\left\{B\right\},0\right],\left[\left\{C\right\},0.005\right],\left[\left\{\Omega \right\},0.5\right]\right)$$

$$\left(E_{2},m_{2}^{\alpha }\right)=\left(\left[\left\{A\right\},0\right],\left[\left\{B\right\},0.495\right],\left[\left\{C\right\},0.005\right],\left[\left\{\Omega \right\},0.5\right]\right)$$

$$\left(E_{m_{1}⊕m_{2}},m_{1}⊕m_{2}\right)=\left(\left[\left\{A\right\},0.3300\right],\left[\left\{B\right\},0.3300\right],\left[\left\{C\right\},0.0067\right],\left[\left\{\Omega \right\},0.3333\right]\right)$$

For decision making in evidence theory, the final BPA after constant fusion can be transformed to a pignistic probability. Such a map from BPA to a kind of probability function is called the pignistic transformation, which is defined as follows [75].

**Definition** **4.** *Assume the frame of discernment is $\Omega =\{A_{1},A_{2},\ldots ,A_{n}\}$, the pignistic probability function is defined as:*

(5) $$BetP\left(A\right)=\sum_{H\subseteq \Omega }\frac{\left|A\cap H\right|}{\left|H\right|}\frac{m(H)}{1-m(\emptyset )},\;\forall A\subseteq \Omega$$

*where |A| is the cardinality of set A.*

### 2.2. The Credibility Decay Model

Song et al. [43] defined a model for dynamic information combination. Assume evidences $e_{1},e_{2},\ldots ,e_{n}$ are collected at n time nodes $t_{1},t_{2},\ldots ,t_{n}$. $m_{1},m_{2},\ldots ,m_{n}$ is a group of BPAs of these evidences on the discernment frame.

Smets [44] gives the requirement of Markovian for data fusion on time series as follows.

**Definition** **5.** *Let $f_{n}\left(m_{1},m_{2},\ldots ,m_{n}\right)$ be the final result of n evidences dynamic combination. $f_{n}$ is qualified as Markovian (see [43,44]) if and only if there is a g function can be defined as [43]:*

(6) $$f_{n}\left(m_{1},m_{2},\ldots ,m_{n}\right)=g\left(g\left(\ldots \left(g\left(g\left(m_{1},m_{2}\right),m_{3}\right),\ldots \right),m_{n-1}\right),m_{n}\right)$$

Markovian requirement improves the computational efficiency of BPAs’ combinations or data fusion for there is no need to store all past BPAs and compute repeatedly.

Evidences collected from sensors at different time nodes are not fully trusted in CDM. α is the credibility used to discount the old evidences every time like Equation (4) shows. So the Equation (6) in CDM can be defined as follows.

**Definition** **6.** *Let $\alpha_{i-1}=\alpha \left(dt\right)=\alpha \left(t_{i}-t_{i-1}\right)$, for i=2,3,…,n. Function g can combine two BPA being defined as [43]:*

(7) $$f_{n}\left(m_{1},m_{2}\ldots ,m_{n}\right)=g\left(g{\left(\ldots {\left(g{\left(g{\left(m_{1}^{\alpha_{1}},m_{2}\right)}^{\alpha_{2}},m_{3}\right)}^{\alpha_{3}},\ldots \right)}^{\alpha_{n-2}},m_{n-1}\right)}^{\alpha_{n-1}},m_{n}\right)$$

Further, the credibility in CDM is defined as follows.

**Definition** **7.** *Let $m_{j}$ be a BPA on the frame of discernment collected at time $t_{j}$, the dynamic credibility at time node $t_{i}\left(i=j+1\right)$ is defined as [43]:*

(8) $$\alpha_{i,j}=e^{-\lambda (t_{i}-t_{j})}$$

*where λ>0.*

In CDM, after the data is first transformed to the BPA on the discernment frame at time $t_{i}$, the dynamic credibility $\alpha_{i,j}$ calculated from Equation (8) is used to discount old BPA according to Equation (4). The whole dynamic data fusion process is Equation (7).

### 2.3. The Ordered Weighted Aggregation Operator

OWA operator receives much attention and has been used in a wide range of applications [76,77] since it was first introduced. Furthermore, this aggregating way is defined as follows:

**Definition** **8.** *Assume there are n criteria $A_{1},A_{2},\ldots ,A_{n}$. A mapping $I^{n}\to I$ (where I∈[0,1]) is called an OWA operator when there is a weight vector $w={\left(w_{1},w_{2},\ldots ,w_{n}\right)}^{T}$ satisfies [50]:*

(9) $$OWA_{w}\left(b_{1},b_{2},\ldots ,b_{n}\right)=\sum_{i=1}^{n}w_{i}b_{i}$$

*where $b_{i}$ is the ith largest element in the collections $a_{1},a_{2},\ldots ,a_{n}$ which meets $A_{1},A_{2},\ldots ,A_{n}$. And the weight vector has the following properties:*

1. $w_{i}\in \left[0,1\right]$
2. *$\sum_{i=1}^{n}w_{i}=1$.*

In [50], the way to generate OWA weight is defined as following:

**Definition** **9.** *Let Q be a nondecreasing proportional fuzzy linguistic quantifiers [50], then the OWA weight satisfies:*

(10) $$w_{i}=Q\left(\frac{i}{n}\right)-Q\left(\frac{i-1}{n}\right),\;i=1,2,\ldots ,n$$

*In [78], Zadeh defined Q as:*

(11) $$Q\left(r\right)=\left\{\begin{array}{cc} 0, & r<a\\ \frac{r-a}{b-a}, & a\leq r\leq b\\ 1, & r>b \end{array}\right.$$

*where a,b,r∈[0,1].*

## 3. A New CDM Based on OWA

**Figure 1:**
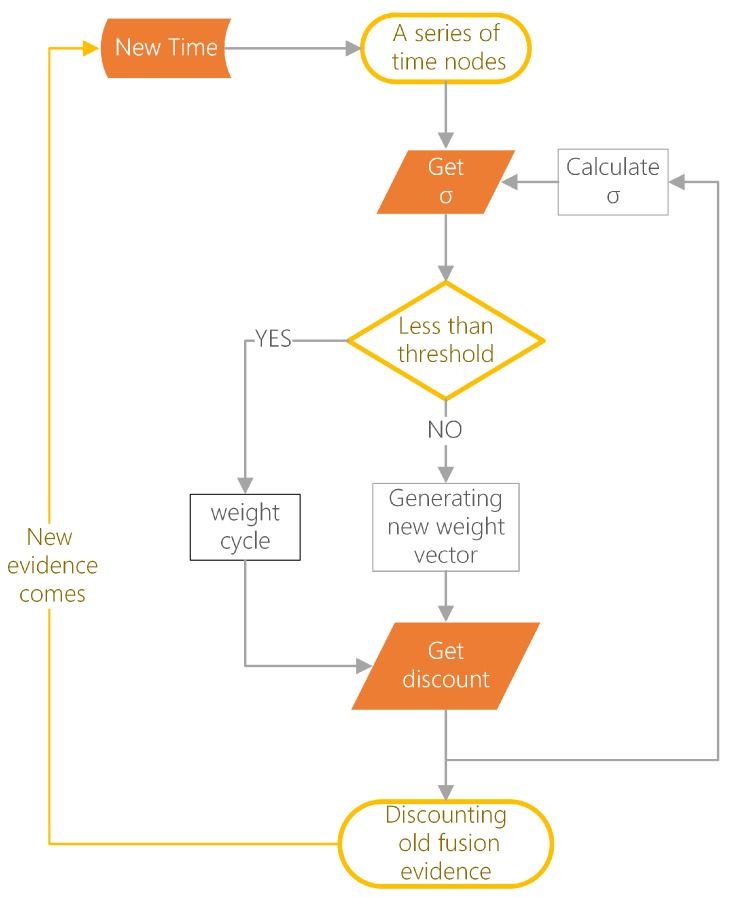
Process to generate OWA discounts.

In this section, a new dynamical generating OWA weights method is proposed to replace Equation (8) in original model. Then using Equations (10) and (11), proper OWA discount weight can be obtained in the new CDM when each new evidence comes. The whole process to generate dynamical discount weights is shown in Figure 1. More details are shown step by step as follows.

Since the OWA operator reorders different satisfaction degrees in descending order, the front weights always have comparatively large demands. Additionally, in time series, a newer time node has a bigger numerical value. So the vector component $w_{1}$ is always the weight for evidence collected at the latest time node *n*. This weight represents the current satisfactory degree to the fusion result got from time node n−1. Similarly, at the earliest time node 1, before the first evidence comes, the system is totally unknown. So weight component $w_{n}$ always represents unknown.

The data fusion based on time series should take timeliness into consideration, which means the effect of old evidences (in fusion form) should gradually reduce. The proposed method evaluates such a degree as follows.

**Definition** **10.** *Assume there are n time nodes $t_{1},t_{2},\ldots ,t_{m},\ldots ,t_{n}$. The measure to evaluate the effect degree of old evidences from time node m is as follows:*

(12) $$\sigma_{m,n}=\prod_{i=m}^{n}\alpha_{i-1,i}$$

*where $\alpha_{i-1,i}$ is the credibility at time $t_{i}$. Additionally, $\sigma_{m,n}$ may represent the effect degree at current time $t_{n}$ from the old evidence collected time $t_{m}$.*

Normally let m=2, which means σ is calculated from time node 2. As proposed, an additional threshold parameter k is used to control discount speed, which means *k* is a threshold and for the first time node $t^{\prime }$ satisfying σ≤k, when new evidences come after $t^{\prime }$, since the effect degree of old evidences has met our demand, reusing weights before $t^{\prime }$ is reasonable. Clearly, *k* with higher value causes higher credibility and lower discount speed to old evidence. When the data source is more reliable which may be judged by other information, the value of *k* can be more higher. Let set *k* equals to 0.01 in this paper.

Based on time series, the proposed method can dynamically generate a weight vector when new evidences comes as follows.

**Definition** **11.** *Assume that we collect a series of evidences from n time nodes with n≥2. Let $m_{i}$ be the BPA of evidence $e_{i}$ collected at time $t_{i}$, for i=1,2,…,n with $t_{i}>t_{i-1}$. Based on n time nodes, the weights can be obtained as follows:*

(13) $$W^{t}=\left[\begin{array}{c} w_{1}^{t}\\ w_{2}^{t}\\ \cdots \\ w_{n}^{t} \end{array}\right]$$

*t is the newest or latest time node. Particularly, $w_{1}$ is the weight of $t_{n}$, and $w_{n}$ is the weight of $t_{1}$.*

In fact, based on time effectiveness and to get more sound fusion information by reducing the influence of interference data, $w_{1}$ is a prior focus.

Since the importance of evidence depends on the new and old level, by using fuzzy linguistic quantifier Q shown in Definition 9, the OWA operator in proposed method can assign satisfaction according to the new and old level of evidences collected from sensors on different time series.

Further more, some properties for the values of *a* and *b* in proposed method shown in Equation (11) are as follows:

1. *a* must equal to 0, otherwise $w_{1}$ may equal to 0.
2. When *b* closes to 0, more satisfaction is given to the newest fusion evidence.
3. When *b* closes to 1, less satisfaction is given to the newest fusion evidence.

**Figure 2:**
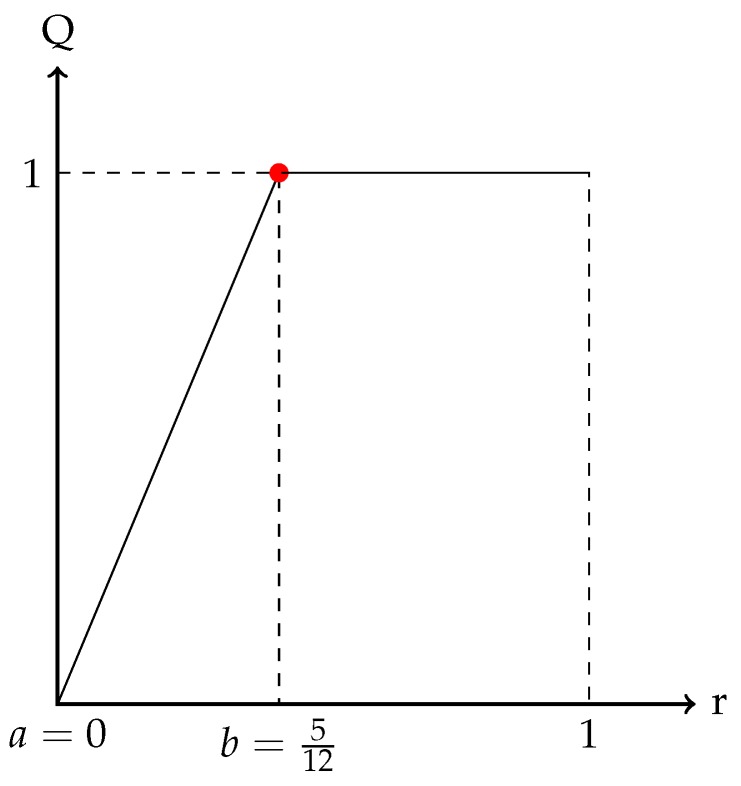
Function Q.

Lets a=0 and $b=\frac{5}{12}$, Q function can be got as Figure 2 shows.

**Example** **2.** *Assume evidences $e_{1},e_{2},e_{3},e_{4}$ and $e_{5}$ are collected from sensors at five time nodes, $t_{1}=1$ s, $t_{2}=10$ s, $t_{3}=20$ s, $t_{4}=22$ s and $t_{5}=25$ s. According to Equations (11) and (10), the weights are as follows:*

$$\begin{array}{ccccc} w_{1}^{t_{2}}=Q\left(\frac{1}{2}\right)-Q\left(0\right)=1 & w_{2}^{t_{2}}=Q\left(1\right)-Q\left(\frac{1}{2}\right)=0\\ w_{1}^{t_{3}}=Q\left(\frac{1}{3}\right)-Q\left(0\right)=\frac{12}{15} & w_{2}^{t_{3}}=Q\left(\frac{2}{3}\right)-Q\left(\frac{1}{3}\right)=\frac{3}{15} & w_{3}^{t_{3}}=Q\left(1\right)-Q\left(\frac{2}{3}\right)=0\\ w_{1}^{t_{4}}=Q\left(\frac{1}{4}\right)-Q\left(0\right)=\frac{12}{20} & w_{2}^{t_{4}}=Q\left(\frac{2}{4}\right)-Q\left(\frac{1}{4}\right)=\frac{8}{20} & w_{3}^{t_{4}}=Q\left(\frac{3}{4}\right)-Q\left(\frac{2}{4}\right)=0 & w_{4}^{t_{4}}=Q\left(1\right)-Q\left(\frac{3}{4}\right)=0\\ w_{1}^{t_{5}}=Q\left(\frac{1}{5}\right)-Q\left(0\right)=\frac{12}{25} & w_{2}^{t_{5}}=Q\left(\frac{2}{5}\right)-Q\left(\frac{1}{5}\right)=\frac{12}{25} & w_{3}^{t_{5}}=Q\left(\frac{3}{5}\right)-Q\left(\frac{2}{5}\right)=\frac{1}{25} & w_{4}^{t_{5}}=Q\left(\frac{4}{5}\right)-Q\left(\frac{3}{5}\right)=0 & w_{5}^{t_{5}}=0 \end{array}$$

$W^{t_{2}}={\left(1,0\right)}^{T}$

$W^{t_{3}}={\left(\frac{12}{15},\frac{3}{15},0\right)}^{T}$

$W^{t_{4}}={\left(\frac{12}{20},\frac{8}{20},0,0\right)}^{T}$

$W^{t_{5}}={\left(\frac{12}{25},\frac{12}{25},\frac{1}{25},0,0\right)}^{T}$

*Clearly, weights have no relation to time interval.*

Example 2 details the satisfaction level with old evidences at different time nodes. For example, the weight vector $W^{t_{5}}$ shows that at time node $t_{5}=25s$, the current old evidences (in fusion form) with $\frac{12}{25}$ satisfaction level need to be given $\frac{12}{25}$ credibility value and the past time nodes’ old evidences are given $\frac{12}{25}$, $\frac{1}{25}$, 0, 0 credibility value respectively. Since the Markovian requirement also need to be satisfied in proposed model like Equations (6) and (7) shows, $w_{1}$ from each weight vector should be given more attention.

**Example** **3.** *Assume that there are 10 evidences $e_{1},e_{2},\ldots ,e_{10}$ collected at $t_{1}$, $t_{2},\ldots ,t_{10}$. The effect degree of old evidences from time node 1 can be calculated as follows:*

$$\sigma_{2,n}=\prod_{i=2}^{n}\alpha_{i-1,i}=\prod_{i=2}^{n}w_{1}^{t_{i}}$$

*So we have:*

$$\sigma_{2,2}=1$$

$$\sigma_{2,3}=1\times 0.8=0.8$$

$$\sigma_{2,4}=1\times 0.8\times 0.6=0.48$$

…

$$\sigma_{2,8}=1\times 0.8\times 0.6\times \ldots \times 0.032=0.0096\leq k=0.01$$

$$\sigma_{2,9}=\sigma_{2,8}\times w_{1}^{t_{2}}=0.0096\times 1=0.0096$$

*More details and results are shown in Table 1.*

In Example 3, after time node 8, since the effect degree of old evidences is less than threshold. Fusion discount is reused from time node 9. The proposed method first ensures that the impact of old evidences falls as quickly as possible to a level less than expectation (0.01), and at this time, reusing $w_{1}$ as credibility to discount old evidences again is reasonable.

## 4. Application

In this section, two applications in target recognition are given with the comparison to original CDM to illustrate the new model.

In the next work, we call the new CDM as OWA model, and let the original model (OM) gets discount from Equation (8) with λ=0.15 [43].

**Example** **4.** *Assume there are three targets A, B, C constructing the discernment frame Ω={A,B,C} and the target A is the right one. The series of evidences $e_{1}$ to $e_{10}$ which support different targets collected from sensors are shown in Table 1. More details are shown in Table 2.*

**Figure 3:**
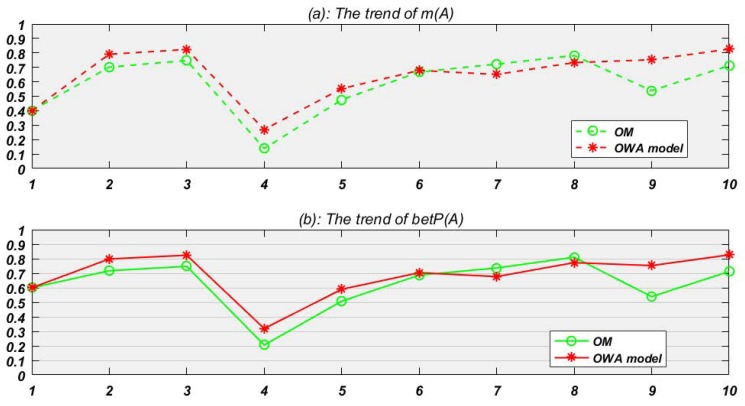
Results of Example 4.

*OM and OWA model are used for data fusion respectively for target identification. The fusion result of m(A) at each time node is shown in Figure 3a. And to identify the most possible target with known information, the pignistic probability trend of target A is obtained according to Equation (4) (Figure 3b).*

In Table 2, BPA collected at $t_{1}$ represents that target A, B, C is given 0.4, 0.1, 0.1 belief respectively, and there is still 0.2 belief which cannot be divided to A or B and another 0.2 belief which also cannot be divided to A or C. Since $t_{1}$ is the first time node, so there is no credibility to old evidence and no fusion happens. Then at $t_{2}$, just like the table shows, the credibility to old evidence (it is $e_{1}$ at this time) in proposed OWA model and OM is 1 and 0.638 respectively.

In most situations from Example 4, the new model presents better than the original one like Figure 3a red line shows, though at time node 7 and 8, OWA model seems do a bit worse. In fact, from Table 1 we can see that the threshold k controls the speed to discount old fusion evidences. Setting a comparative bigger numerical value for k can lower the decay speed and make discounts enter the next weight cycle quicker. Normally, the evidence source is more reasonable, the decay speed is slower.

To further study the identification rate of two methods in Example 4, the BPA is transformed to the pignistic probability to make decision. For example, the BetP of A, B, C at $t_{1}$ is 0.6, 0.2, 0.2 according to Equation (5) as follows.

$$BetP\left(A\right)=1\times m\left(A\right)+\frac{1}{2}\times m\left(AB\right)+\frac{1}{2}\times m\left(AC\right)=0.6$$

$$BetP\left(B\right)=1\times m\left(B\right)+\frac{1}{2}\times m\left(AB\right)=0.2$$

$$BetP\left(C\right)=1\times m\left(C\right)+\frac{1}{2}\times m\left(AC\right)=0.2$$

**Figure 4:**
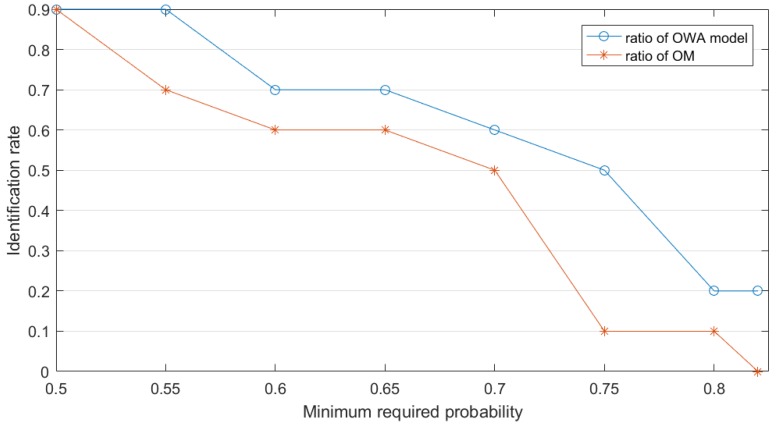
Identification effect in Example 4.

Then we can set the minimum requirement probability for sensors to make a decision which means when a target has met the probability, which can be thought correct. We can set the identification probability equal to 0.5 at first. The identification rate means that if BetP(A)>0.5, the sensors can conclude that the unknown target is A, which also means that at this time node the target is identified correctly. Note that at decision making happens at every time node. By gradually upgrading the minimum requirement for sensors’ judgment, we always find that the identification rate to the right target A (shown in Figure 4) of proposed OWA model is better than original model.

Example 4 dimly shows the shortcoming for OM. At time node 9, since the evidence comes only a bit slow, OM fails to converge to a sound decision. The next example further studies this kind of situation.

**Figure 5:**
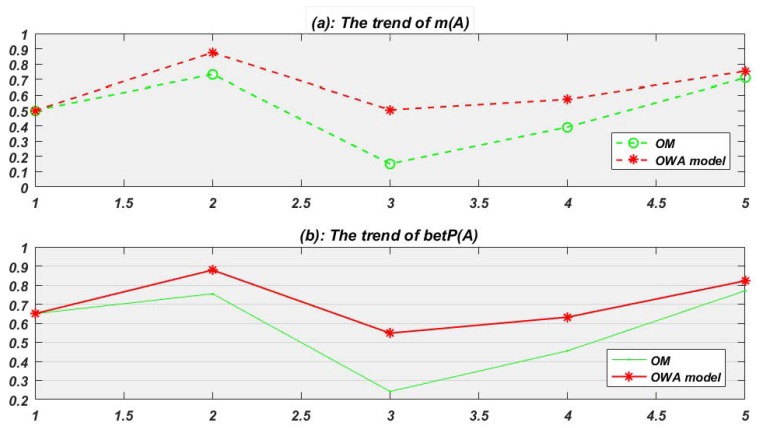
Results of Example 5.

**Example** **5.** *Assume there are three targets A, B, C constructing the discernment frame Ω={A,B,C} and the target A is the right one. There are 5 time nodes from sensors, they are shown in Table 3. With the change of time, the BPA changes of the right target A is shown in Figure 5a,b*.

**Figure 6:**
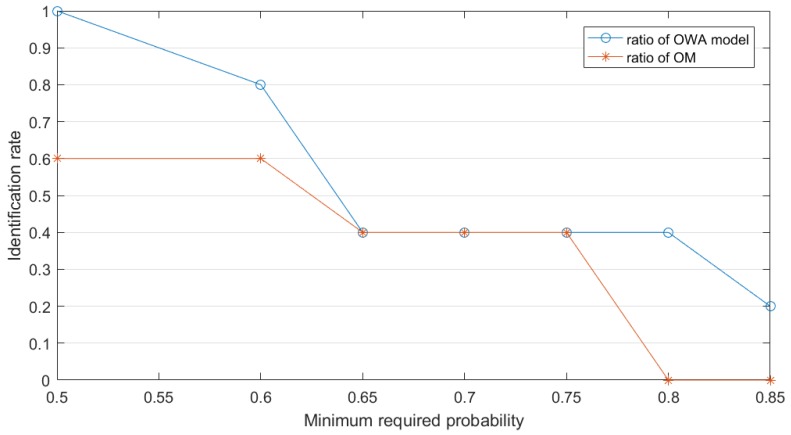
Results of Example 5.

The identification rate of A in Example 5 is obtained and shown in Figure 6. Compared with performance of the OM in the figure, the proposed method clearly has a higher or identical identification rate to hit the right target A from $t_{1}$ to $t_{5}$.

Example 5 also shows that OM is associated to time interval too much. Time node 3, 4 and 5 show the drawbacks in OM clearly. When bad information comes slowly, the original model recovers toughly. At this time, if reliable evidences come a little slowly as well, its recovery becomes much tougher except very high sound and correct evidences constantly happens. Another problem is that a short time interval may aggravate the effect of interference and slow down the converging speed like time node 3 and 4 show.

Clearly, new model with OWA weight as discount performs better in both situations, for it devotes attention to decay degree itself instead of time intervals. It can also resist interference and keep converging speed by reusing weights according to Equation (12).

## 5. Conclusions

How to combine time series data is still an open issue. To overcome the shortcomings of existing credibility decay model, a new method based on OWA operator is presented. OWA weights based on series of time nodes are used to substitute for discount function in original model. After the application in target recognition, it is explicit that new CDM can do better than the original one and adapt to various situations as well. One advantage of our proposed method is that the time interval is reasonably considered. The proposed method has the promising aspects in time series data fusion.

## Figures and Tables

**Table 1:** Weights and *σ*.

Time Node	$w_{1}^{t_{i}}$(Credibility α)	$\sigma_{2,n}$(Effect Degree)
$t_{1}$ = 1 s	-	-
$t_{2}$ = 4 s	1.000	1.000
$t_{3}$ = 7 s	0.800	0.800
$t_{4}$ = 17 s	0.600	0.480
$t_{5}$ = 20 s	0.480	0.230
$t_{6}$ = 23 s	0.400	0.092
$t_{7}$ = 25 s	0.343	0.032
$t_{8}$ = 29 s	0.300	0.009
$t_{9}$ = 39 s	1.000	0.009
$t_{10}$ = 42 s	0.800	0.002

**Table 2:** 10 evidences with BPAs and their discounts.

$t_{i}$	m(A)	m(B)	m(C)	m(AB)	m(AC)	m(BC)	m(Ω)	α in OWA Model	α in OM
$t_{1}$ = 1 s	0.4	0.1	0.1	0.2	0.2	0	0	-	-
$t_{2}$ = 4 s	0.6	0.2	0.1	0	0.05	0.05	0	1	0.638
$t_{3}$ = 7 s	0.65	0.15	0	0	0	0.2	0	0.8	0.638
$t_{4}$ = 17 s	0.1	0.75	0	0.15	0	0	0	0.6	0.223
$t_{5}$ = 20 s	0.6	0.3	0	0.1	0	0	0	0.48	0.638
$t_{6}$ = 23 s	0.65	0.25	0	0	0	0	0.1	0.4	0.638
$t_{7}$ = 25 s	0.6	0.3	0	0	0	0	0.1	0.343	0.741
$t_{8}$ = 29 s	0.7	0.2	0	0.1	0	0	0	0.3	0.549
$t_{9}$ = 39 s	0.5	0.5	0	0	0	0	0	1	0.223
$t_{10}$ = 42 s	0.65	0.1	0.15	0	0	0.1	0	0.8	0.638

**Table 3:** BPAs from sensors in Example 5.

Time Node	BPA	α in OWA Model	α in OM
$t_{1}$= 1 s	m(A) = 0.5, m(B) = 0.1		
	m(C) = 0.1, m(AB) = 0.2	-	-
	m(AC) = 0.1		
$t_{2}$= 10 s	m(A) = 0.7, m(B) = 0.1		
	m(C) = 0.1, m(AC) = 0.05	1.00	0.259
	m(BC) = 0.05		
$t_{3}$= 20 s	m(A) = 0.1, m(B) = 0.7	0.80	0.223
	m(AB) = 0.2		
$t_{4}$= 22 s	m(A) = 0.5, m(B) = 0.2	0.60	0.741
	m(ABC) = 0.3		
$t_{5}$= 30 s	m(A) = 0.7, m(B) = 0.1	0.48	0.638
	m(AB) = 0.2		
